# Histone Modifications, Internucleosome Dynamics, and DNA Stresses: How They Cooperate to “Functionalize” Nucleosomes

**DOI:** 10.3389/fgene.2022.873398

**Published:** 2022-04-28

**Authors:** Wladyslaw A. Krajewski

**Affiliations:** N.K. Koltsov Institute of Developmental Biology of Russian Academy of Sciences, Moscow, Russia

**Keywords:** nucleosomes, histones, hexasomes, ubiquitylation, histone modifications, histone code, DNA stresses

## Abstract

Tight packaging of DNA in chromatin severely constrains DNA accessibility and dynamics. In contrast, nucleosomes in active chromatin state are highly flexible, can exchange their histones, and are virtually “transparent” to RNA polymerases, which transcribe through gene bodies at rates comparable to that of naked DNA. Defining mechanisms that revert nucleosome repression, in addition to their value for basic science, is of key importance for the diagnosis and treatment of genetic diseases. Chromatin activity is largely regulated by histone posttranslational modifications, ranging from small chemical groups up to the yet understudied “bulky” ubiquitylation and sumoylation. However, it is to be revealed how histone marks are “translated” to permissive or repressive changes in nucleosomes: it is a general opinion that histone modifications act primarily as “signals” for recruiting the regulatory proteins or as a “neutralizer” of electrostatic shielding of histone tails. Here, we would like to discuss recent evidence suggesting that histone ubiquitylation, in a DNA stress–dependent manner, can directly regulate the dynamics of the nucleosome and their primary structure and can promote nucleosome decomposition to hexasome particles or additionally stabilize nucleosomes against unwrapping. In addition, nucleosome repression/ derepression studies are usually performed with single mononucleosomes as a model. We would like to review and discuss recent findings showing that internucleosomal interactions could strongly modulate the dynamics and rearrangements of nucleosomes. Our hypothesis is that bulky histone modifications, nucleosome inherent dynamics, internucleosome interactions, and DNA torsions could act in cooperation to orchestrate the formation of different dynamic states of arrayed nucleosomes and thus promote chromatin functionality and diversify epigenetic programming methods.

## Introduction

Many diseases and behavioral pathologies such as cancer (Espinosa, 2008; Cao and Yan, 2012; Johnsen, 2012; Cole et al., 2015), metabolic disorders (Gluckman et al., 2009; Gao et al., 2014), cardiovascular and autoimmune diseases, and diabetes (Dieker and Muller, 2010; Zou et al., 2014) are the results of gene deregulation (Gray, 2006; Perini and Tupler, 2006; Bhaumik et al., 2007; Weake, 2014; Mirabella et al., 2016). However, despite the critical importance of gene regulatory principles for the diagnosis, prevention, and therapy of genetic diseases (and, in general, for directed manipulation of gene activity), many aspects of gene regulation have not been well-elucidated thus far and remain unclear on how DNA processing machineries overcome the tight multilevel packaging of DNA in cell nuclei.

**FIGURE 1:**
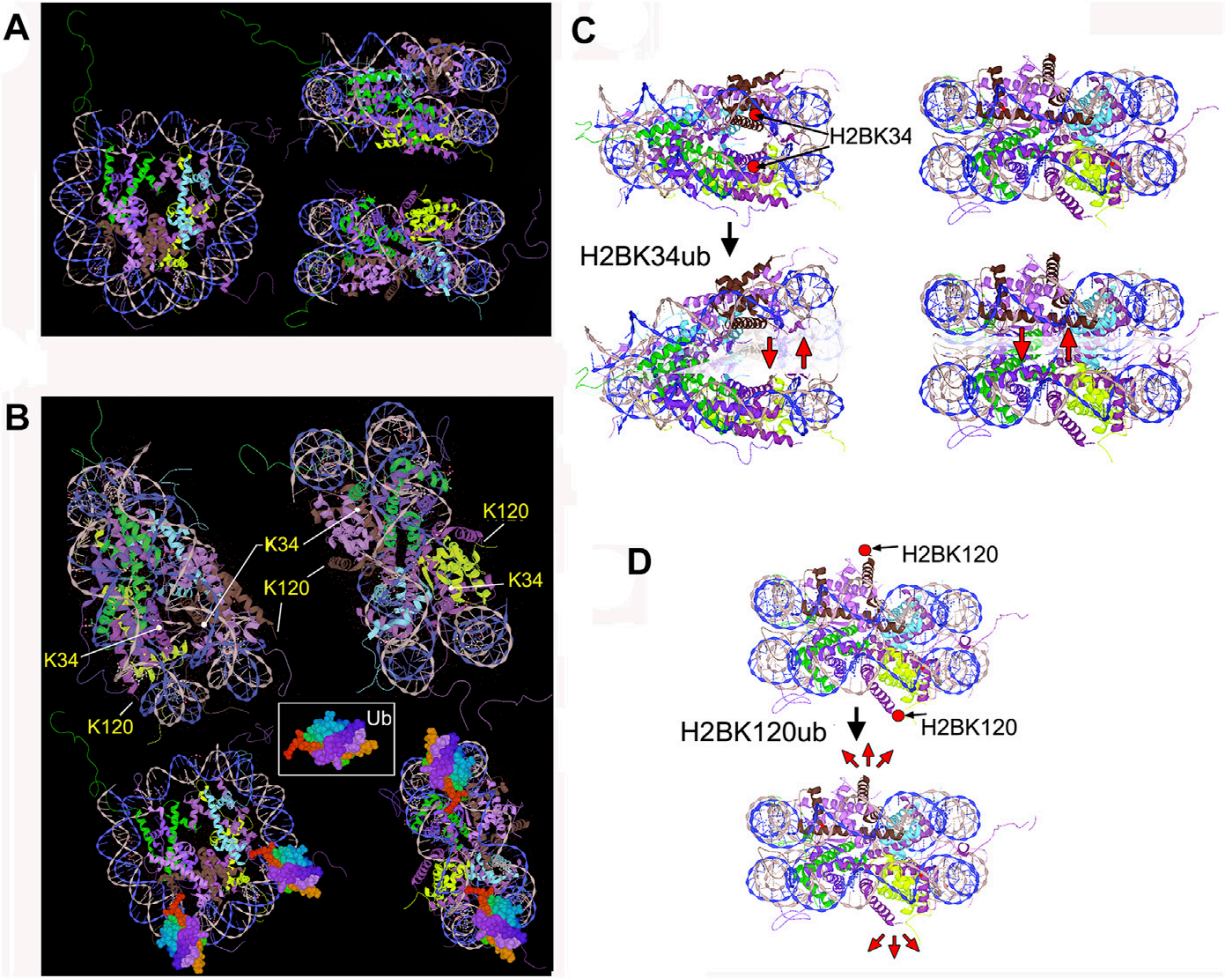
**(A)** Nucleosome (1kx5) front, top, and side view. •• H3.2 (chains A,E), •• H2B 1.1 (chains D,H), •• H2A type 1 (chains C,G), and •• H4 (chains B,F) **(B)** Positions of H2B K34 and K120 indicated by arrows. Drafts at the bottom illustrate the H2BK34ub nucleosome (Ubiquitin PDB: 1ubq). **(C,D)** Sketches, depicting the potential mechanisms of nucleosome-destabilizing effects by H2BK34-/ K120-ubiquitylation. **(C)** H2BK34ub installed in the occluded nucleosome region could act as a “wedge”, facilitating DNA gyre–gyre separation (“gaping”). **(D)** Mechanistic forces applied to the H2A-H2B dimer by ubiquitin deposited to H2B termini could weaken the nucleosome through stochastic motions of bulky ubiquitin and/or its steric clashes with the nucleosome surface. This could also promote nucleosome breathing and DNA-dimer opening motions.

In eukaryotes, the genetic information required to control all life processes exists in the form of chromatin, a complex hierarchical structure of DNA super-helices, stabilized by a multitude of protein–DNA and protein–protein interactions. On the first level of compaction, 147 bp of every ∼200 bp of DNA are wrapped in 1.75 turns around an octamer of histone proteins, comprising one H3-H4 tetramer flanked on each side by H2A-H2B dimers (Figure 1A), thus forming nucleosomes, the basic repeated chromatin units (Luger et al., 1997; Vasudevan et al., 2010). Nucleosome arrays fold into ‘solenoids (Kruithof et al., 2009; Kepper et al., 2011; Victor et al., 2012) or “zig-zag”-like (Dorigo et al., 2004; Grigoryev, 2004; Schalch et al., 2005; Grigoryev et al., 2009) arrangements to form the 25–34 nm chromatin fiber, stabilized by linker histones H1/H5 (Robinson and Rhodes, 2006). The 30-nm fiber further self-associates and condenses into higher-order tertiary structures.

Nucleosomes in their “canonic” state (as seen by X-ray studies (Luger et al., 1997; Richmond and Davey, 2003; Vasudevan et al., 2010) are rather robust static units, refractory to DNA-binding proteins, and thus, literary should present an “immovable barrier” even for the “irresistible force” of progressing RNA polymerases (Kornberg and Lorch, 1991). So, despite the nucleosomes being the key elements in gene regulation (Gibney and Nolan, 2010), it is still understudied how they relieve their intrinsically repressive effects on DNA expression.

Due to the structural tensions associated with the bending of the stiff (Manning, 2006) 147 bp core nucleosomal DNA around the histone globule, nucleosomes retain some degree of dynamicity and undergo spontaneous fluctuations of nucleosome wrapping, which range from 10–250 ms “breathing” and more slow “opening” motions (Koopmans et al., 2007; Armeev et al., 2018), up to the lo 1–10 min nucleosome hinge-like “gaping” openings (Zlatanova et al., 2009; Ngo and Ha, 2015). Fluctuations in nucleosome wrapping and transiently increasing DNA exposure (Polach and Widom, 1996) play important role in regulating the accessibility of transcription factors to the nucleosome DNA (Li et al., 2005) and alleviating RNA polymerases entering the nucleosome (Hodges et al., 2009; Selth et al., 2010). It is possible that any stimuli increasing the basic level of inherent nucleosome dynamics will contribute to the derepression of the nucleosome.

Nucleosomal histones are subjected to a multitude of reversible posttranslational modifications (PTMs) which, supposedly, control virtually all aspects of chromatin functioning. According to the “histone code” concept, PTMs “acting in a combinatorial or sequential fashion on one or multiple histone tails specify unique downstream function” (Strahl and Allis, 2000). Many PTMs are dynamically deposited during the cell cycle to control particular cellular processes, whereas certain histone PTMs are thought to program the transcription memory transmitted to the progeny cells. A different (though debatable (Alabert et al., 2015; Reveron-Gomez et al., 2018)) view that histone PTMs are not transmitted to progeny chromatin but instead persistently bound histone-modifiers reestablish the PTM pattern on the daughter chromatin (Petruk et al., 2012; Petruk et al., 2013) came from histone H3K4me3/ H3K27me3 “inheritance” studies, which used (probably insufficiently sensitive) proximity-ligation assay to monitor the modified histones on replicated DNA. Finally, several recent studies (Reinberg and Vales, 2018; Escobar et al., 2019; Escobar et al., 2021) provided evidence that the repressive histone modifications but not active ones are inherited upon DNA replication.

In a classic view, both “small” (methylation and acetylation) (Rothbart and Strahl, 2014) and “bulky” (ubiquitin and SUMO-1,2/3 polypeptides (Cubenas-Potts and Matunis, 2013; Weake, 2014)) histone PTMs are considered binding targets for effector proteins (Rothbart and Strahl, 2014; Andrews et al., 2016) or as regulators of chromatin higher-order folding (acting by modulation of internucleosome interactions (Pepenella et al., 2014b; Prakash and Fournier, 2018)) but not as direct triggers of primary nucleosome structure reversing DNA repression. By tuning histone charges and deposition of a modest steric bulk, “small” histone PTMs only moderately affect the spontaneous fluctuations of nucleosomes without affecting their stability (PTMs at the nucleosome entry-exit) or modestly decrease nucleosome stability without affecting nucleosome dynamics (PTMs near the nucleosome dyad axis) (Bowman and Poirier, 2015; Armeev et al., 2018).

However, recent data suggest that currently understudied large polypeptide PTMs could play an active role in directly altering the nucleosome primary structure and dynamics. In addition, since intrinsic chromatin organization is based on a hierarchy of DNA helices (DNA double helix, nucleosome DNA wrapping, and chromatin fiber), the chromatin structure is subjected to superhelical stresses in DNA, which could significantly affect the nucleosome properties and functionality. Furthermore, due to multiple internucleosome interactions, the model describing a nucleosome array just as a polymer of individual “canonic” nucleosomes does not adequately recapitulate nucleosome functionalities. The interaction between nucleosomes *via* flexible histone termini could significantly affect nucleosome structural transitions (Krajewski, 2016). We would like to discuss these phenomena in view of the recent and older literature data.

## Nucleosomes as Key Elements in Epigenetic Regulation of Chromatin Activity

What differentiates nucleosomes in transcriptionally active chromatin from the canonic ones? In both cases, these particles possess the same composition and share the same organizational principles. However, in transcribed chromatin regions, nucleosomes are dynamic and (Zlatanova et al., 2009; Armeev et al., 2018) exhibit high conformational flexibility (Saavedra and Huberman, 1986; Morse et al., 1987; Krajewski and Luchnik, 1991), easily exchange their histone subunits (Zlatanova et al., 2009; Venkatesh and Workman, 2015), and support fast progression of RNA polymerases (Singh and Padgett, 2009) that is accompanied by nucleosome unfolding and unshielding of histone H3 sulfhydryls which are otherwise buried at the nucleosome dyad and inaccessible in the canonic nucleosome state (Prior et al., 1983; Chen et al., 1991).

A notable hallmark of the transcribed chromatin is the dynamic monoubiquitylation of histone H2B at lysines K120 (K123 in yeast) (Batta et al., 2011; Fleming et al., 2008; Trujillo and Osley, 2012; Wright and Kao, 2015) and K34 (Li et al., 2017; Wu et al., 2011; Wu et al., 2013; Wu et al., 2014) (Figure 1B). This feature would be consistent with a series of recent findings showing that K34-ubiquitylation of histone H2B (and H2BK120ub to a lesser degree) can significantly enhance nucleosome dynamics, decrease nucleosome stability, and promote eviction of one histone H2A-H2B dimer (Krajewski et al., 2018; Krajewski W. A., 2020), especially in the presence of histone chaperons. This effect is likely due to the steric hindrances by “bulky” ubiquitin moieties, which destabilize the nucleosome (Krajewski, 2019; Krajewski WA., 2020). The resulting hexasome particle was stable, suggesting that dissociation of one ubiquitylated histone dimer is sufficient to relieve the steric stresses incurred by massive ubiquitin moieties (Krajewski et al., 2018; Krajewski W. A., 2020).

The 8.6 kD ubiquitin (Renatus et al., 2006) and 10–12 kD SUMO (Bayer et al., 1998; Huang et al., 2004) are close in size to histones that principally distinguishes these PTMs from “small” chemical modifications. A steric bulk deposited by ubiquitylation and sumoylation could act to “mechanically” alter the canonic nucleosomes. Nucleosome-destabilizing forces would be stronger when bulky PTMs are deposited within the nucleosome lateral surface and so, directly conflict with the compact nucleosome structure. For example, ubiquitylation of histone H2B at lysine K34, which is “buried” between two DNA gyres (Li et al., 2017; Wu et al., 2011) (Figure 1B), could act as a “wedge”, facilitating DNA gyre–gyre opening (Figure 1C). Bulky PTMs at histone termini (e.g., H2BK120ub, Figure 1B) disturb the nucleosome core less but could affect intra-nucleosomal interactions, for example, by electrostatic repulsion (Figure 1D). The association of H2A-H2B dimers on the nucleosome interface could be weakened by stochastic (Brownian) motions of the attached PTMs, which will tend to “tear-off” one histone dimer out from the nucleosome interface (Figure 1D). Of note, it has been shown that dynamic nucleosome conformations could be shifted to more unwrapped structures by binding bulky objects to the nucleosome periphery (Polach and Widom, 1996; Buning et al., 2015), such as the transcription factors (Polach and Widom, 1996), an adjacent nucleosome, or long linker DNA (Buning et al., 2015). Due to the interactions between histone tails and nucleosome-associated core DNA (Cutter and Hayes, 2015; Shaytan et al., 2016; Chakraborty and Loverde, 2017; Morrison et al., 2018) or linker DNA (Davey et al., 2002; Cutter and Hayes, 2015; Schunter et al., 2017), bulky PTMs of histone termini could destabilize the intra-nucleosome interactions either directly or by colliding with the nucleosome surface.

These results suggest a hypothesis (Krajewski, 2019; Krajewski WA., 2020) that in contrast to “small” histone PTMs, attachment to nucleosomes at certain positions of ubiquitin (and, supposedly, other bulky PTMs) could potentially represent an *in vivo* mechanism to functionalize canonic nucleosomes by strikingly increasing their dynamics and triggering the conversion of a nucleosome to a more functionally active hexasome particle.

Interestingly, recent single-molecule magnetic tweezer experiments (Xiao et al., 2020) have shown that H2AK119ub, on the contrary, dramatically prevents the peeling of the DNA from the histone octamer that stabilizes the nucleosome. The stabilizing effect of ubH2A was not a result of the enhanced stability of the octamer (ibid) but likely relies on the Ub-mediated steric clashes that prevent nucleosome unfolding. Although these results would benefit from refinement with more relevant biochemical approaches, it could be supposed that at some nucleosome positions, “hindrances” caused by bulky modifications could strongly stabilize and “lock” the nucleosome unwrapped state. With this example, one can propose that “bulky” modifications could create a stable “code” of both active and repressed chromatin states.

Histone ubiquitylation is one of the key epigenetic marks with a wide spectrum of action (Weake, 2014), so the functions of H2BK120ub and H2BK34ub (and H2A119ub) are not only limited to the proposed “direct” nucleosome-regulatory role but also involve other ubiquitylation-mediated binding events for the chromatin regulators (Vaughan et al., 2021). There are still less data available on H2BK34ub; therefore, we will just mention here two recent studies on the interactions of Dot1L and H2BK120ub nucleosomes, which are critical to direct H3K79 methylation (Anderson et al., 2019; Valencia-Sanchez et al., 2019).

Previous work showed that H2B-ubiquitylation is sufficient to directly enhance the nucleosome dynamics and nucleosome-hexasome transition *in vitro* (the effects were comparable to those produced by ATP-driven chromatin remodelers) and, supposedly, *in vivo*. But, however, the “direct” and “indirect” (*via* other regulatory factors) nucleosomal effects of the bulky PTMs are not self-exclusive. There also might be an interplay between histone ubiquitylation and another histone PTMs and their corresponding co-factors regulating chromatin dynamics *in vivo*. One example is that PRC2 co-factors JARID2 and AEBP2 play a crucial role in both the recruitment and activation of PRC2 through their recognition of H2AK119ub1 (Kasinath et al., 2021), which further orchestrates the local chromatin environment.

**FIGURE 2:**
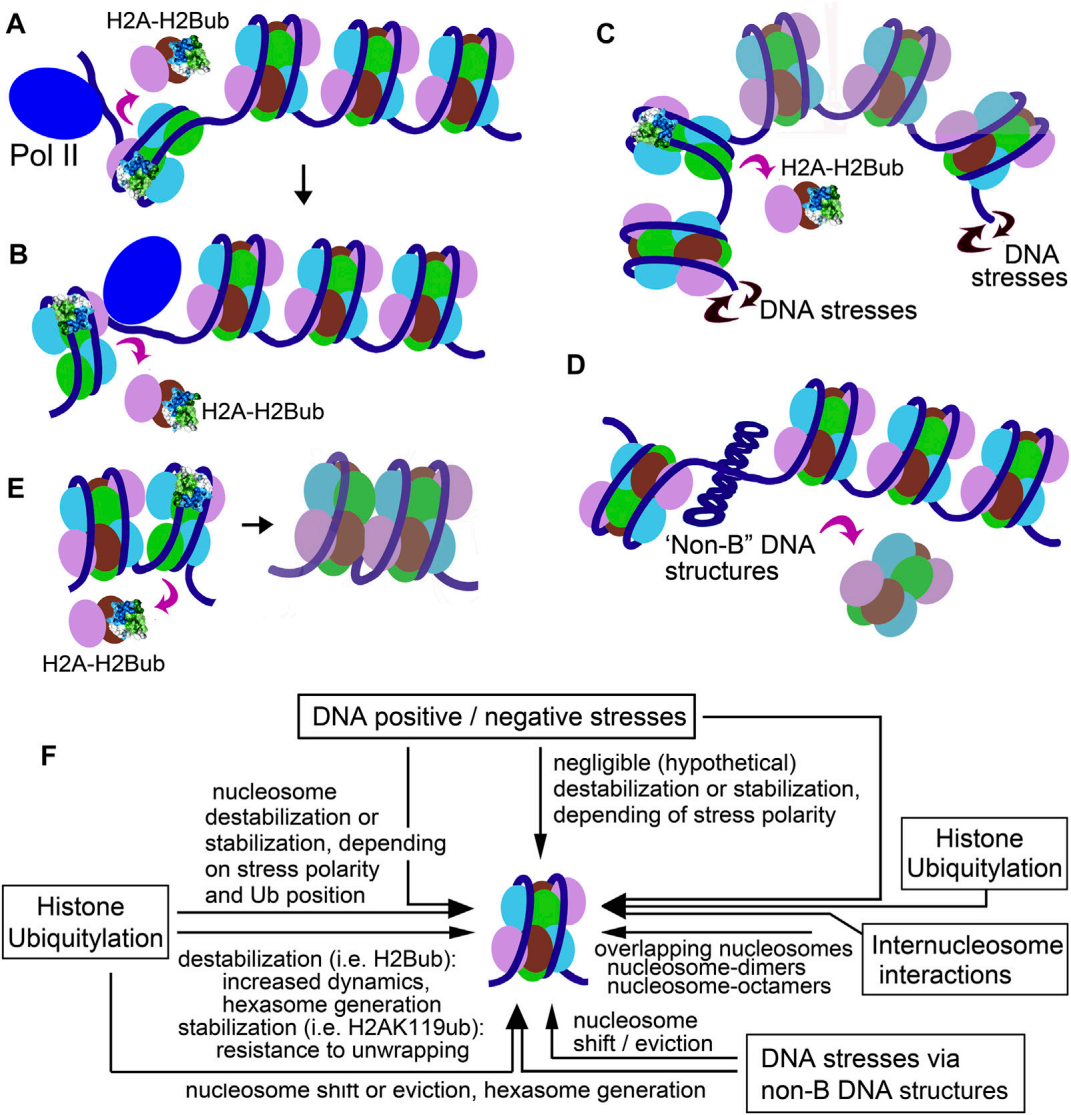
Sketches illustrating the nucleosomal effects of H2Bub. **(A,B)** RNA Pol II traversing the H2B-ubiquitylated nucleosome. **(A)** Eviction of the promoter-proximal H2A-H2B dimer promotes the polymerase complex to enter the nucleosome; **(B)** Eviction of the promoter-distal H2A-H2B dimer promotes RNA Pol II to successfully elongate through the nucleosome. **(C)** Hexasome generation by DNA stresses. **(D)** Nucleosome rearrangement by transition of a DNA segment to a cruciform structure. **(E)** Formation of “overlapping” nucleosomes. **(F)** Schematic figure, depicting how different mechanisms (histone ubiquitylation, DNA stress, and internucleosome interaction) could cooperate to regulate chromatin dynamics and function.

The tight link of H2BK120/ K34-ubiquitylation with transcription and replication shows a plausible mechanism assisting RNA and DNA polymerases to overcome the nucleosome barrier. The MOF–MSL complex, which deposits H2BK34ub (Krajewski and Vassiliev, 2019; Wu et al., 2011), plays a critical role in transcription, initiation, and elongation and is enriched at transcription start sites (Wu et al., 2014). Regardless of the exact mechanism, it could be hypothesized that H2BK34ub-facilitated destabilization and a dimer eviction in +1 nucleosome (which presents a greater transcription barrier *in vivo* than downstream nucleosomes (Adelman and Lis, 2012; Gilmour, 2009; Teves and Henikoff, 2014; Weber et al., 2014)) assists transient uncoiling of the promoter-proximal boundary of the +1 nucleosome and facilitates the release of Pol II from pausing and its transition to elongation step (Figures 2A,B). PAF1 associated with MOF–MSL and RNF20/40 (which deposit H2BK120ub (Hwang et al., 2003)) progresses together during transcription and elongation (Wu et al., 2014) that supposes that H2B-ubiquitylation, in cooperation with histone chaperones (Hsieh et al., 2013; Hsieh et al., 2015; Gurova et al., 2018), orchestrates unwrapping/rewrapping of transcribed nucleosomes by facilitating coordinated sequential dissociation and rebinding of the nucleosome-proximal and nucleosome-distal H2A-H2B dimer—steps required for RNA Pol II to traverse the nucleosome (Kulaeva et al., 2013). In contrast, H2AK119ub (associated with silenced genes (Meas and Mao, 2015)) could prevent Pol II progression and block remodeling activities (i.e., Swi-Snf and related) that act through peeling on DNA from the nucleosome.

In general, the consequences of histone ubiquitylation and sumoylation on the nucleosome primary structure are still understudied, although the experimental data support the direct, destabilizing, or stabilizing effects of bulky PTMs. *In vitro* H4K34-monoubiquitylation moderately destabilizes nucleosomal association of the H3–H4 tetramer, supposedly, due to the clash between DNA phosphate backbone and deposited ubiquitin (Machida et al., 2016). *In vivo* H3K4-polyubiquitylation by RNF8 promotes nucleosome disassembly and eviction from the DNA (Xia et al., 2017), although it is not clear whether this could be a direct effect of histone ubiquitylation. UV-irradiation activates the ubiquitylation of histones H3 and H4 by CUL4-DDB, promoting the eviction of histones and stimulating the recruitment of XPC repair protein (Wang et al., 2006). In biochemical studies, H2AK119-monoubiquitylation had marginal nucleosome stabilizing/ destabilizing effects (Fierz et al., 2012) but could directly alter the nucleosome interface *in vivo* and protect the H3K36 residue from modification (Bi et al., 2016). Using single-molecule magnetic tweezers, it has been shown that H2AK119ub stabilizes the nucleosome from unwrapping (Xiao et al., 2020) (see above).

There is less data on histone sumoylation. In yeast cells, genetically engineered multiple sumoylation of histone H2B had only a minor structural effect on nucleosomes (Chandrasekharan et al., 2009). The H4K12su is a gene silencing marker (Shiio and Eisenman, 2003; Nathan et al., 2006), despite the H4K12 position being near the H4 basic patch where the steric bulk and hindrances by installed SUMO polypeptides could affect the critical (for chromatin compaction) interaction between H4 tails and the H2A-H2B acidic patch on the adjoining nucleosome (Allahverdi et al., 2011; Pepenella et al., 2014b). Indeed, spFRET studies have shown that H4K12su destabilizes long-range internucleosome interactions and moderately represses the formation of compact chromatin (Dhall et al., 2014).

## DNA Stresses and DNA Non-Canonic Structures in Epigenetic Control

Virtually any process that manipulates DNA strands can generate positive or negative DNA torsional stress (Esposito and Sinden, 1988; Baranello et al., 2012; Gilbert and Allan, 2014; Corless and Gilbert, 2016). For example, waves of positive and negative supercoiling are generated ahead and behind the RNA polymerase, respectively (Liu and Wang, 1987), which may be directly observed *in vivo* (e.g., (Lee and Garrard, 1991; Ljungman and Hanawalt, 1995; Naughton et al., 2013; Gerasimova et al., 2016)) and *in vitro* (Pfaffle and Jackson, 1990; Jackson, 1993; Bancaud et al., 2007). It is assumed that negative DNA stresses favor DNA wrapping on the histone octamer, while positive supercoiling destabilizes nucleosomes (Esposito and Sinden, 1988; Pfaffle and Jackson, 1990; Clark et al., 1993; Jackson, 1993; Bancaud et al., 2007). It would be appealing to attribute to DNA stresses an active role in the regulation of a primary nucleosome structure. Indeed, the generation of artificially high levels of positive DNA torsions in a single chromatin fiber by magnetic tweezers can break histone dimer–tetramer docking and induce transient, reversible nucleosome reorganization (Bancaud et al., 2007); the authors assume that a wave of such nucleosome chiral transitions can propagate ahead of a transcribing polymerase *in vivo*. However, after decades of studies, there is still no consensus whether the “physiological” levels of DNA torsions under physiologically relevant conditions could have any substantial effect on the nucleosome structure. Both supporting (Garner et al., 1987; Jackson, 1993; Sheinin et al., 2013; Teves and Henikoff, 2014) and opposing (Clark et al., 1993; Sheinin et al., 2013) observations were published. The “physiological” levels of DNA supercoiling only marginally affected the stability of unmodified nucleosomes *in vitro*—such that histone octamers assembled on negatively supercoiled DNA with only a slight preference compared to that of positively supercoiled DNA (Clark and Felsenfeld, 1991; Clark et al., 1993). This question is of particular importance since years of studies accumulated mounting evidence of how cells could regulate DNA stresses. In addition, numerous “non-canonical” DNA structures have been discovered, which are capable of adopting non-B DNA conformation to absorb or enhance DNA torsions (Smith, 2008; Baranello et al., 2012; Kaushik et al., 2016).

A different situation could be if a nucleosome structure is already intrinsically destabilized by deposited bulky histone modification. We propose that DNA topology, favoring or disfavoring nucleosome wrapping, may contribute to the structural effects of histone ubiquitylation (Krajewski, 2019; Krajewski WA., 2020) (Figure 2C). In our experiments, “physiological” negative and positive supercoiling in long DNA templates had opposing (stimulating or inhibitory, respectively) effects on the hexasome generation upon assembly of H2BK34ub nucleosomes (Krajewski et al., 2018) but had no effect on unmodified nucleosomes. We suppose that nucleosome “unfolding” using moderate positive DNA stress restrains the steric hindrances in ubiquitylated nucleosomes, while nucleosome compaction by negative stresses enhances the hindrances (Krajewski et al., 2018). More strong DNA topology effects in short (298 bp) minicircle DNAs have diverse effects on unmodified, H2BK34ub and H2BK120ub nucleosomes (Krajewski, 2018), suggesting that DNA topology states can strongly and selectively (and, likely, bi-directionally) affect nucleosome stability and dynamics depending on the type of H2B-ubiquitylation. It is notable that certain DNA topologies increased the stability of H2BK120ub nucleosomes over unmodified ones (see (Xiao et al., 2020) and discussion above). The H2BK34- and H2BK120-ubiquitylated nucleosomes exhibited quite selective sensitivity and sustainability to positive and negative DNA stresses (Krajewski, 2018; Krajewski et al., 2018), implying that bulky PTMs could play an active role in amplifying or mitigating the nucleosomal effects of DNA torque (including those by translocating RNA Pol II) and, thus, highlighting the nucleosome-regulatory role of DNA stresses. It could be interesting to see how positive and negative DNA stresses could affect “DNA-peeling refractory” H2AK119ub nucleosomes.

In addition to their direct nucleosome stability effects, DNA stresses could also affect the nucleosomes “indirectly” by generating non-standard DNA structures. Even relatively short stretches of alternating (CG) pairs and inverted repeat DNA sequences can form different structural isomers (left-handed helices and cruciforms) in response to superhelical stress at low “physiological” densities (Esposito and Sinden, 1988; McLean and Wells, 1988; Smith, 2008; Wells, 1988). These structures can regulate (absorb) superhelical stresses in DNA and also can affect nucleosome distribution by “translationally shifting” histone octamers along with DNA or displacing nucleosomes from the DNA (Figure 2D). Many studies suggest that Z-DNA and cruciforms cannot be organized in the nucleosome. Deposition of nucleosomes on supercoiled DNA containing a region of Z-DNA or a cruciform leads to the exclusion of regions of Z-DNA from the interiors of nucleosome cores *in vitro* and *in vivo* (Krajewski, 1996).

## Internucleosome Interaction as an Additional Source of Chromatin Functionality

A “nucleosome-octamer” and “nucleosome-dimer” structure in which a nucleosome particle is associated with an additional histone octamer (Voordouw and Eisenberg, 1978; Stein, 1979; Daban and Cantor, 1982; Ausio et al., 1984; Aragay et al., 1988; Aragay et al., 1991) or another nucleosome (Tatchell and Van Holde, 1979; Ausio et al., 1984; Yager et al., 1989), respectively, was described years ago, although since then was forgotten for decades. Both the nucleosome-octamers and nucleosome-dimers are likely to be formed *via* trans-interactions between histone octamers. The site-directed histone-DNA and histone–histone cross-linking (Zheng and Hayes, 2003a; Zheng and Hayes, 2003b; Kan et al., 2007; Kan and Hayes, 2007; Kan et al., 2009; Pepenella et al., 2014a) revealed multitude interactions between histone tails and DNA of neighboring nucleosomes (reviewed in: (Luger et al., 2012; Pepenella et al., 2014b; Krajewski, 2016)). The nuclease digestion pattern and digestion kinetics of nucleosome-octamers and nucleosome-dimers are similar to those in single nucleosomes; therefore, it could be supposed that these particles largely retain the basic features of nucleosomal organization (Stein, 1979; Eisenberg and Felsenfeld, 1981; Krajewski and Vassiliev, 2012).

The ability of a nucleosome to bind extra histone octamers/dimers could play an important gene regulatory role during transient chromatin disassembly–reassembly through DNA replication or transcription. For example, a nucleosome behind the RNA Pol II could transiently bind a histone octamer or the evicted histone H2A/H2B dimer from the nucleosome being transcribed—this could be a possible mechanism of how the nucleosome reinstates its initial position on the DNA after the passage of the RNA Pol II complex.

The interaction between nucleosomes could, supposedly, affect chromatin remodeling and deposition of histone modifications. In polynucleosomes, human and yeast Swi/Snf complexes can generate structurally altered ‘asymmetric’ pairs of adjacent nucleosomes (Ulyanova and Schnitzler, 2005; Krajewski and Vassiliev, 2010). These “autosome” structures contain intact histone core octamers, but their nuclease cleavage pattern indicates the association of one internucleosomal and one subnucleosomal (220 and 70 bp, respectively) DNA fragment. In dinucleosomes, Isw1a/b and Isw2 generate extra structural alterations compared to mononucleosomes (Krajewski, 2013; Krajewski, 2014). Remodeling of the nucleosome-dimer particles by yeast Isw2 facilitated *in vitro* the association of nucleosome-dimers with the MLL SET-domain polypeptide (Krajewski and Vassiliev, 2012). SET7 and ALL-1 SET polypeptides showed binding preferences for dinucleosomes (but not mononucleosomes) remodeled with yIsw1/Isw2. The assembly of nucleosomes in oligonucleosomes promoted histone H3 methylation by the EZH2/EED, which only inefficiently modifies single mononucleosomes (Martin et al., 2006). Furthermore, reorganization of di- and oligonucleosomes (but not mononucleosomes) by binding of histone H1 further increased H3 methylation by EZH2 (Martin et al., 2006). However, there is no direct evaluation of the significance of internucleosomal interactions in promoting increased PRC2 HMTase activity as of yet. It could be that dinucleosome-enhanced PRC2 HMTase activity is largely due to the mechanism of allosteric activation (Jiao and Liu, 2015; Yu et al., 2019), and incorporation of H1 further facilitates positioning and activity of the PRC2 complex (that is indirectly supported by strong inhibition of methylation with over-stoichiometric amounts of H1 (Martin et al., 2006)). In general, the reports showed that adjusting the internucleosome spacing could affect the activity of the writers of histone PTMs including PRC2, but many of these studies were performed in an artifactual manner by changing the nucleosome spacing length.

It could be supposed that spontaneous movements of nucleosomes along the DNA, nucleosome dynamic fluctuations, and nucleosome instability incurred by histone ubiquitylation, even in absence of chromatin remodeling activities, could result in transient relocation of a histone H2A-H2B dimer from one nucleosome to the surface of the neighboring nucleosome, thus facilitating the formation of hexasomes and other subnucleosomal structures. Similarly, the hexasome particle generated by histone ubiquitylation could transiently associate with the adjacent nucleosome to form the structurally altered “autosome-like” arrangement. Owen-Hughes’ lab has shown that interactions between two nucleosomes could generate partial unwrapping of one nucleosome with the eviction of one H2A/H2B dimer and “merging” the resulting hexasome and a nucleosome into a single particle in which overlapping octamers and hexasomes invade each other’s space (Engeholm et al., 2009). The authors supposed (ibid) that nucleosome overlapping could be promoted by the eviction of H2A-H2B dimer and by exposure of the nucleosome DNA-binding surfaces. Engeholm et al. supposed that this could occur by the action of Swi-/Snf-related remodeling activities, which can reduce the stability of nucleosomal association of the histone dimer (Bruno et al., 2003; Vicent et al., 2004) and unravel up to 50 bp from the edge of the nucleosomes (Fan et al., 2003; Flaus and Owen-Hughes, 2003; Kassabov et al., 2003; Krajewski and Vassiliev, 2010), such that the nucleosomes may associate through the exposed DNA-binding surfaces to form dinucleosome-like particle (Schnitzler et al., 2001; Ulyanova and Schnitzler, 2005; Ulyanova and Schnitzler, 2007). It is possible that other pathways resulting in destabilized binding of histone dimers with the nucleosome and promoting hexasome generation, such as histone ubiquitylation and nucleosome-destabilizing DNA stresses, could facilitate nucleosome colliding and overlapping (Figure 2E).

## Conclusion

Here, we tried to briefly overview the evidence showing that cooperation between bulky histone modifications, DNA stresses, DNA non-canonic structure, and internucleosomal interactions could create an additional “layer” of chromatin activity determinants Figure 4E. We hypothesize that in such manner, these factors could create a “code” of chromatin activity states, in addition to the histone code of chromatin activity signals, which could promote the formation and stabilization of a highly dynamic, accessible structure of a nucleosome array. The proposed models stress the diversity of mechanisms by which histone PTMs, DNA conformations, and internucleosomal interactions regulate chromatin functionality.

## Data Availability

The original contributions presented in the study are included in the article/Supplementary Materials, further inquiries can be directed to the corresponding author.
